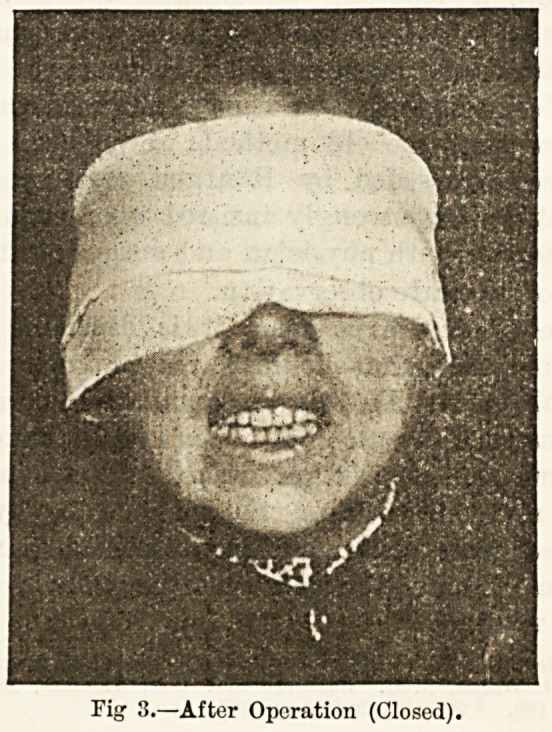# A Case of Ankylosis of Both Temporo-Maxillary Joints

**Published:** 1899-10-07

**Authors:** J. Jackson Clarke

**Affiliations:** Surgeon to Out-patients at the North-West London and City Orthopædic Hospitals.


### A CASE OF ANKYLOSIS OF BOTH TEMPORO-MAXILLARY JOINTS.

By J. Jackson Clarke, M.B.Lond., F.R.C.S., Surgeon to Out-patients at the North-West London and City Orthopædic Hospitals.

Obliteration of the temporo-maxillary joint is not of uncommon occurrence, and it may arise in various ways, *e.g.*, from traumatism, such as dislocation and fracture of the jaw, or penetrating wounds; from suppuration, whether of a pyæmic character, as after scarlet and other fevers; from gonorrhœa and other forms of suppuration; by extension from middle-ear suppuration. Complete osseous ankylosis has been observed as the result of tubercular disease. Virtual ankylosis from deformation of the articulating surfaces, accompanied by disappearance of the inter-articular fibro-cartilage, is not infrequently met with in rheumatoid arthritis. Another form of virtual ankylosis is that due to cicacatricial changes in the soft parts external to the joint; the latter condition, together with the spasmodic fixation of the jaw observed in young adults, and due to defective eruption of the wisdom teeth, are to be carefully distinguished from ankylosis due to intra-articular changes.

As an example of true ankylosis, arising in inflammation, probably of a pyæmic character, and its successful surgical treatment, I will narrate the following example:—

Mrs. S., aged 32, was admitted to the North-West London Hospital under my care on April 22nd of this year.

*Family History.*—Patient's mother died of phthisis; father, of apoplexy; grandfather, of "rheumatic gout."

*Personal History.*—Patient was married in 1899. The first child was born a year later, and died of heart failure at the age of nine months. The second child died shortly after labour. Both labours were difficult, forceps being used.

*Previous Illness.*—After the second pregnancy, patient two and a half years ago was in a provincial hospital for a "tear about the womb" and leucorrhœa. An operation was performed, and within two days the left knee became swollen and painful; it was incised and drained. Soon afterwards the temporo-maxillary joints became painful, and eventually the knee and jaw became fixed. Patient was in hospital for four months. Some time after leaving hospital the knee was straightened under chloroform, and the joint again became swollen and painful. Patient has had no power of movement in the lower jaw for the last two and a-half years.

*Present Condition.*—Patient is somewhat emaciated. The movement of the lower jaw is limited to the power of opening the mouth to the extent shown in Fig. 1, which is a reproduction of a photograph taken just before the operation described below. The masseters are but slightly wasted. The left knee is stiff and immovable, and in a condition of genu valgum. Patient's tongue is furred, and the breath offensive from impaired digestion.

Fig. 1.—Before Operation.

*Operation.*—Chloroform was administered by Dr. L. A. Parry. Under anæsthesia I found that, short of using such a degree of force that would threaten fracture of the jaw, I was unable to break down the adhesions in the joints, or to open the mouth any more widely than the patient had been able to do. I made an incision through the skin over the lower border of the posterior two-thirds of the zygoma of the right side, and having deepened the incision over the uppermost limit of the joint blunt hooks were inserted to draw the branches of the facial nerve forwards, and the auriculo-temporal nerve backwards. The incision was then deepened in the whole of its extent, and the back part of the origin of the masseter detached. I then made a vertical skin-incision, passing from the horizontal incision over the middle of the joint for three quarters of an inch. A blunt hook was inserted to draw down the facial nerve, and then the incision was carried down to the neck of the jaw, which I then cleared by blunt dissection, and passed an aneurism needle behind it in order to draw a piece of silk attached to a Gighli's saw behind the neck of the jaw. The latter was then sawn through close to the condyle, and the removal of the whole of the neck of the bone was completed by dividing the bone again half an inch lower down by combined use of the chisel and bone forceps. About half an inch of bone was thus removed. I then repeated the operation on the left side, using a key-hole saw, chisel, and bone forceps to effect the removal of bone. The jaw was now freely movable, and the wounds in the skin were sewn up throughout their whole extent.

April 25th.—The patient expressed a feeling of relief from the previous feeling of fixity of the jaw.

April 26th.—Some pain was experienced on the left side, and two stitches being removed about ʒii of serum escaped.

April 27th.—Patient very comfortable.

April 29th.—Patient very comfortable. Liquid diet, eggs, milk, &c., still adhered to on account of pain experienced in mastication. The tongue is still much furred, and breath offensive, in spite of free actions of the intestine.

May 1st.—Patient sleeps well. No pain.

May 3rd.—Dressings and sutures removed. Wounds healed throughout. Patient can open the mouth well and widely, and close it again completely.

May 5th.—Patient has now no pain on movement of the jaw. She cannot advance the jaw quite as far as normal owing to severance of the jaw from the attachment of the external plerygoid muscles. The patient now gets up and feels well. Tongue cleaner.

May 11th.—Patient left the hospital. She is now able to enjoy solid food, and can talk comfortably.[1]

The illustration Fig. 2 represents the patient's capacity of opening, and Fig. 3 that of closing, the jaw at the present time, four and a half months after operation. The power of protruding the jaw has been partially regained. This I attribute to certain details

Fig. 2.—After Operation (Open).

of orthopædic after-treatment, consisting in the patient's performing passive movements twice daily. The patient has completely recovered her health, the digestion is normal, and there is no discomfort in masticating solid food.

*Comments.*—The inflammatory process that led to ankylosis in this case was in all probability of an infective character, starting in some suppuration of the genital passages. The danger of forcible correction of ankylosis when due to suppuration is illustrated by the sequel of the operation performed on the left knee; and I have little doubt that a similar mode of treatment had it been applied to the lower jaw would have been equally unsuccessful. My original idea was to break

Fig 3.—After Operation (Closed).

down the adhesions under anæsthesia and then to proceed to the removal of the condyles of the jaw. I found, however, that no force that could be safely used was capable of breaking down the adhesions, so I altered the plan to removal of the neck of the bone on each side. As regards the operation itself, care is required to avoid injury to the important structures that are in close relation to the joint. By using Gighli's saw on the right, and the usual instruments on the left side I was able to form an opinion of the value of the former, which is a length of twisted steel wire ending in a loop at each end for the attachment of a handle. It acts as an universal fret-saw. The section it made was much cleaner than that made by the chisel and forceps, and since it cuts from within out it is worked with perfect security. In future, when I have occasion to perform this operation, I should divide the bone almost completely with Gighli's saw at the upper and the lower limits of the neck and then complete the division by means of the forceps. I may add that simple section of the neck of the bone has been found to be insufficient in these cases.

[1] For the above notes I am indebted to Mr. M. C. Hayward, late Senior Resident M.O. at the North-West London Hospital.

## Figures and Tables

**Fig. 1. f1:**
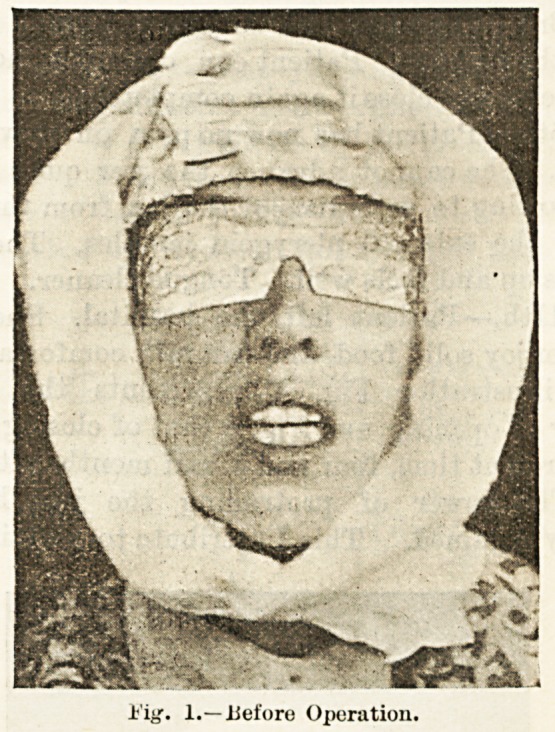


**Fig. 2. f2:**
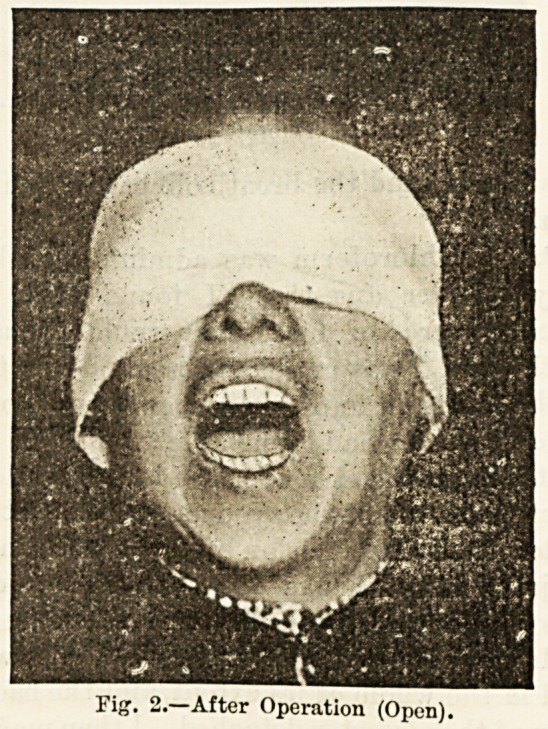


**Fig 3. f3:**